# Nontechnical Skills (NTS) and the Quality of Conducting Prehospital Advanced Cardiopulmonary Resuscitation Among Paramedics

**DOI:** 10.1155/emmi/2207053

**Published:** 2026-06-07

**Authors:** Tomasz Ilczak, Michał Ćwiertnia, Kacper Sumera, Piotr Babik, Piotr Białoń, Mieczysław Dutka, Iwona Malinowska-Lipień, Magdalena Augustyn, Mateusz Majewski, Anna Lis, Piotr Leszczyński, Arkadiusz Stasicki, Piotr Abramczyk, Paweł Kukla, Wioletta Pollok-Waksmańska, Joanna Trojak-Piętka, Marek Kawecki

**Affiliations:** ^1^ Department of Emergency Medicine, Faculty of Health Sciences, University of Bielsko-Biala, Bielsko-Biala, Poland, bielsko.pl; ^2^ European Pre-Hospital Research Network, Nottingham, UK; ^3^ East Midlands Ambulance Service NHS Trust, Nottingham, UK; ^4^ Department of Biochemistry and Molecular Biology, Faculty of Health Sciences, University of Bielsko-Biala, Bielsko-Biala, Poland, bielsko.pl; ^5^ Faculty of Health Sciences, Jagiellonian University Medical College, Krakow, Poland, cm-uj.krakow.pl; ^6^ Emergency Department, Leszek Giec Upper-Silesian Medical Centre of the Medical University of Silesia in Katowice, Katowice, Poland; ^7^ Department of Medical and Health Sciences, University of Siedlce, Siedlce, Poland; ^8^ Department of Internal Medicine, Hypertension and Vascular Diseases, Medical University of Warsaw, Warsaw, Poland, wum.edu.pl; ^9^ Department of Public Health, Faculty of Health Sciences, University of Bielsko-Biala, Bielsko-Biala, Poland, bielsko.pl

**Keywords:** advanced life support (ALS), cardiopulmonary resuscitation (CPR), nontechnical skills (NTS), paramedic education, soft skills training

## Abstract

**Introduction:**

It is common knowledge that the correct application of cardiopulmonary resuscitation (CPR) requires technical skills such as defibrillation, high‐quality chest compressions, and efficient airway management. However, scientific research is increasingly underlining the role of appropriate training in nontechnical skills (NTS).

**Research Aim:**

This exploratory study aimed to assess the relationship between NTS and the quality of advanced CPR among paramedics.

**Materials and Methods:**

The research involved 51 paramedics randomly assigned to 17 three‐person teams. Each team participated in a 15‐min cardiac arrest scenario. After the first session, the teams were divided into two groups: the intervention group (Group 1), which underwent specialized NTS training, and the control group (Group 2), which did not receive the initial training. Directly after the training phase, all teams from both groups carried out a second attempt during a simulated sudden cardiac arrest (SCA) scenario identical to the first one.

**Results:**

The lowest CPR result in the intervention group (Group 1) was Min = −3.00, and the highest Max = 13.00, while in the control group (Group 2), the lowest result was Min = −42.00, and the highest Max = 8.00 (*p* < 0.05). In Group 1, a statistically significant correlation (*p* < 0.05) was noted between the change in the NTS score and the change in the CPR result. A higher NTS score was accompanied by a higher CPR score.

**Conclusions:**

A short NTS training session was associated with improved NTS application by paramedic resuscitation teams. Furthermore, higher chest compression quality positively correlated with NTS proficiency.

## 1. Introduction

Nontechnical skills (NTS), including team communication and the management of tasks and priorities, are increasingly recognized as critical determinants of the quality of advanced life support (ALS). Despite decades of research, the precise relationship between technical proficiency and nontechnical performance during resuscitation remains poorly defined. [1–5]. It is widely established that strict adherence to ALS guidelines requires proficiency in core technical competencies, including manual defibrillation (incorporating correct electrode placement and safety protocols), high‐quality chest compressions, and advanced airway management [6]. Furthermore, an expanding body of scientific evidence emphasizes the critical impact of structured NTS training on clinical performance and patient safety [7]. Beyond team composition, equipment availability, and technical proficiency, it is essential to identify additional variables that influence the overall quality of ALS interventions [8]. While communication and leadership are recognized as pillars of effective resuscitation, their practical optimization and integration into high‐stress clinical environments often remain a significant challenge [9], notwithstanding their documented impact on the quality of ALS interventions and, consequently, on survival outcomes following sudden cardiac arrest (SCA) [10]. Issues described in the literature, including closed‐loop communication, task prioritization, and continuous situational reassessment, alongside clinical composure, optimize individual procedural performance. By enhancing both the precision and efficiency of interventions, these factors directly correlate with improved survival outcomes. The matrix was created to improve the conduct of advanced resuscitation after applying such skills [11]. The present study objective was to adapt and evaluate the NTS assessment matrix—standardized by the Polish and European Resuscitation Councils—for objective scientific analysis. We hypothesized that the structured implementation of specific communication strategies and medical emergency team (MET) management protocols would yield a quantifiable improvement in cardiopulmonary resuscitation (CPR) quality, specifically regarding chest compression, defibrillation, and advanced airway management. The primary objective of this exploratory study was to assess the relationship between NTS proficiency and the quality of ALS among paramedics, specifically focusing on chest compressions, defibrillation latency, and airway management. We hypothesized that targeted NTS training would lead to a significant improvement in the resuscitation quality index, a reduction in the time to first defibrillation, and faster instrumental airway clearance compared to a control group.

## 2. Materials and Methods

### 2.1. Study Design and Setting

Assessment of the relationship between NTS and the quality of advanced CPR as evaluated by interventions such as chest compressions, defibrillation, and airway management among paramedics. This research was designed as an exploratory, randomized simulation‐based study. The research was conducted in a laboratory accredited by the Polish Accreditation Bureau with accreditation number AB 1701 in the research field of emergency medical intervention. A Laerdal mannequin equipped with QCPR® software was used in the research, as it was considered to be of sufficient quality to assess the correctness of CPR [12]. A module of chest compressions was used that enabled evaluation of compression depth, frequency of pressure, the duration of compression and relaxation phases, and the correct positioning of the hands. The results were presented as a general percentage value calculated on the basis of the software algorithm, illustrating the quality of the resuscitation. In addition, the time of defibrillation was also measured, counted in seconds from the moment the cardiac arrest mechanism was identified, while the time to airway clearance was measured in seconds from the beginning of the scenario. Validated and calibrated stopwatches were used for the measurements (SN: RF‐SW‐140 designation 0931 and SN: RF‐SW‐140 designation 940).

### 2.2. Participants and Eligibility Criteria

The study group comprised paramedics working in the State Medical Emergency System in the Silesian Voivodeship. Initially, 56 people registered to take part in the research and completed informed consent forms for participation in the research, including information on the possibility of video recording. The criteria for inclusion in the research were as follows: (1) a paramedic diploma at the bachelor level, or in emergency medical techniques; (2) at least 5 years of work experience in emergency medical teams or in a hospital emergency department; (3) a completed ALS course no earlier than 3 years before joining the research; and (4) a documented training course for paramedics as the legal basis for working in the emergency medical system in Poland. After verification, five people were excluded from the research as they did not meet the criteria, i.e., 3 people did not have a valid ALS certificate, and the remaining 2 did not have sufficiently long work experience necessary to take part in the research. Ultimately, 51 people qualified for the research out of 56 applications.

### 2.3. Randomization and Allocation Concealment

The group of research participants was then randomly divided into three‐person resuscitation teams, creating 17 teams. For randomization purposes, the https://www.randomizer.org/ application was used, available as freeware. The application generates sequences of pseudorandom numbers independently of the researchers. The list of participant identification numbers was entered into a program that randomly assigned individuals to teams using simple randomization (without blocking or stratification). The randomization was performed by an independent person who did not participate in the further stages of the study, in order to reduce the risk of selection bias.

### 2.4. Study Protocol and Intervention

Each of the teams was led through a 15‐min cardiac arrest scenario in which the mechanism for cardiac arrest was ventricular fibrillation (VF) throughout the scenario. After the first session, the teams were assigned to two groups at random using the described application—an intervention group (Group 1) and a control group (Group 2). After randomization, Group 1 consisted of nine resuscitation teams, while the control group (Group 2) consisted of eight teams. The procedure was conducted by an independent person to maintain the principles of allocation concealment. The intervention group (Group 1) was then trained by a certified GIC instructor in the use of NTS. The training consisted of a multimedia presentation with the use of the NTS matrix presented during certified ALS courses. The training lasted four academic hours. Directly after the training, all teams (from Groups 1 and 2) conducted a second attempt during a simulated SCA scenario identical to the first attempt. The research consisted of two stages. In the first stage, the NTS of the participants in Groups 1 and 2 were assessed during resuscitation procedures.

### 2.5. Outcome Measures and Assessment Tools

To assess the level of NTS, the table of NTS by Cooper et al. [8] was applied in accordance with ERC guidelines. This table is also used to assess the work of teams during certified ALS courses. In accordance with the methodology and teaching program conducted on ALS courses certified by the European Resuscitation Council (ERC) and the Polish Resuscitation Council (PRC). The original version of the NTS matrix consisted of 11 items. However, for the purpose of this study, one item pertaining to “team morale” was excluded. While morale is a vital component of long‐term clinical collaboration, it was deemed to have limited construct validity for a high‐intensity, 15‐min acute care simulation. In order to avoid subjective assessment, the research was conducted each time by the same two ALS and Generic Instructor Course (GIC) instructors, and assessed independently. Points awarded for individual actions depended on whether the assessed element was present during resuscitation. In the second stage, directly after the scenario, the assessment team conducted a debriefing and analyzed the performance of the individual assessed elements. The information was recorded on specially prepared assessment cards. For the assessments, use was made of the points in the Cooper et al. matrix [13] relating to team management, teamwork, and task management, containing the questions: Does the team communicate effectively using verbal and nonverbal communication? Does the team work together to complete the tasks in the relevant time? Does the team act with composure and control? Does the team adapt to changes in the situation? Does the team monitor and reassess the situation? The minimum number of points a team could obtain was 0, while the maximum was 10 points. While the scenario was being conducted in the laboratory, two laboratory technicians were present, whose task was to assess the remaining interventions and to measure the times of defibrillation and airway clearance.

### 2.6. Reliability and Blinding (Formerly Laboratory Technicians)

In order to obtain reliable results, the research assessments were conducted exclusively by ERC‐certified ALS instructors with full qualifications to conduct training, while the two people assessing NTS were also qualified as GIC instructors. The certificate numbers are provided in the additional material. The scenarios were recorded to enable later validation and to confirm the obtained measurements for possible later display when there is uncertainty in the assessment.

### 2.7. Statistical Analysis

Analysis of the research was conducted in the R statistical environment ver.3.6.0, PSPP software, and MS Office 2019. To analyze the quantitative variables presented according to group, Student’s parametric test was applied or its nonparametric equivalent, the Mann–Whitney U‐test. In order to assess the existence of a relationship between the variables, the Pearson’s correlation coefficient or Spearman’s rank correlation coefficient was used. Test selection was carried out on the basis of variable distribution, verified using the Shapiro–Wilk test. The adopted significance level was *p* < 0.05.

### 2.8. Ethical Considerations

The study protocol was reviewed and received a positive opinion from the Research Ethics Committee of the University of Bielsko‐Biala (Approval No. 2021/12/8E/9, dated 2021‐07‐12). All participants provided informed written consent prior to their inclusion in the study. To adhere to the ethical principle of beneficence, members of the control group were offered the opportunity to participate in the same specialized NTS training session following the completion of the data collection period.

## 3. Results

For the entire study group consisting of 17 resuscitation teams, the change was calculated for each parameter of the conducted interventions, that is, the difference between the results for the first and second attempts. Positive values indicate an improvement in the CPR results, a shortening of the time to the first defibrillation, a shortening of the time for airway clearance, and an improvement in the NTS points (Table 1).

**TABLE 1:** Descriptive statistics for interventions conducted by all teams participating in the research.

Variable	*N*	*M*	*SD*	Min	Max	Me
CPR result attempt 1	17	82.53	11.46	60.00	97.00	84.00
CPR result attempt 2	17	83.18	19.09	36.00	97.00	94.00
CPR change	17	0.65	13.37	−42.00	13.00	4.00
1st defibrillation attempt 1	17	32.71	37.86	10.00	160.00	15.00
1st defibrillation attempt 2	17	22.06	30.35	7.00	132.00	11.00
1st defibrillation change	17	10.65	17.07	−12.00	49.00	4.00
Airway clearance attempt 1	17	239.82	111.61	118.00	511.00	197.00
Airway clearance attempt 2	17	191.82	88.57	96.00	385.00	165.00
Airway clearance change	17	48.00	85.40	−178.00	241.00	43.00
NTS attempt 1	17	7.06	2.08	3.00	10.00	7.00
NTS attempt 2	17	7.88	1.69	5.00	10.00	8.00
NTS change	17	0.82	2.30	−5.00	4.00	1.00

*Note: N*—number; *M*—mean; Min—minimum; Max—maximum; Me—median.

Abbreviation: *SD*—standard deviation.

In the intervention group (Group 1), the change in CPR score was not lower than Me = 9.00 for half of it (among the other half of the intervention group, it was not higher than Me = 9.00). The lowest score in this group was Min = −3.00, and the highest was Max = 13.00. In the control group (Group 2), the change was not greater for half of the participants; among the other half, it was lower than Me = −1.00 (among the other half, it was not lower than Me = −1.00). The lowest result was *Min* = −42.00, while the highest was *Max* = 8.00. The research showed that people in the intervention group (Group 1) achieved a statistically significant (*p* < 0.05) greater improvement in the CPR result in relation to people from Group 2. In the intervention group (Group 1), the change in the first defibrillation time was on average *M* = 2.33 (*SD* = 6.80), while in the control group (Group 2), the average was higher, at *M* = 20.00 (*SD* = 20.60). In the control group (Group 2), a statistically significant (*p* < 0.05) greater improvement was achieved in the first defibrillation time. Among half the study group, the change in the airway clearance time was not greater than *Me* = 29.00. The lowest result in this group was *Min* = 6.00, and the highest was *Max* = 177.00. Among half the control group (Group 2), this change was not lower than *Me* = 57.50. The lowest result was *Min* = 6.00, and the highest *Max* = 241.00. The indicated differences are not statistically significant differences (*p* > 0.05), which means that belonging to a particular group did not result in a significant change in the airway clearance time. In the intervention group (Group 1), the NTS change was on average *M* = 1.56 (*SD* = 1.33), and in the control group (Group 2), the average was lower, at *M* = 0.00 (*SD* = 2.93). The indicated differences were not statistically significant (*p* > 0.05). Belonging to a particular group did not therefore result in a significant change in the NTS result (Table 2).

**TABLE 2:** Statistically significant differences for individual assessed parameters depending on the study and the control groups.

	*t/U*	*df*	*p*	Descriptive statistics				
				*M*	*SD*	Min	Max	Me
CPR change	12.50		0.026					
Intervention group (1)	U					−3.00	13.00	9.00
Control group (2)						−42.00	8.00	−1.00
1st defibrillation change	2.32	8	0.048					
Intervention group (1)	t			2.33	6.80			
Control group (2)				20.00	20.60			
Clearance change	29.00		0.531					
Intervention group (1)	U					6.00	177.00	29.00
Control group (2)						−178.00	241.00	57.50
NTS points change	−1.44	15	0.171					
Intervention group (1)	t			1.56	1.33			
Control group (2)				0.00	2.93			

*Note: M*—mean; Me—median; Min—minimum result; Max—maximum result; *p*—statistical significance; t—test statistic; *U*—test statistic.

Abbreviations: *df*—degrees of freedom; *SD*—standard deviation.

**FIGURE 1:**
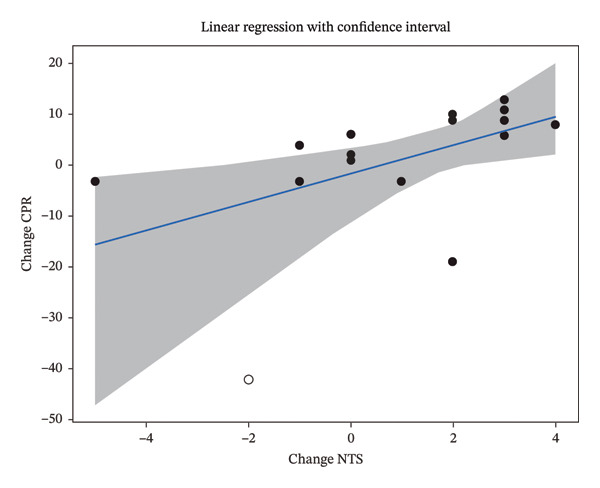
Correlation between the change in NTS score and the change in CPR score total.

The research showed a statistically significant correlation (*p* < 0.05) for the whole study group between the change in NTS points and the change in CPR result. The greater the improvement in the NTS score, the higher the CPR result obtained. However, there was no statistically significant correlation (*p* < 0.05) between the change in the number of NTS points and the change in the 1st defibrillation time or the airway clearance time (Table 3). To illustrate the change in CPR score, the correlation is also presented in Figure 1.

**TABLE 3:** Impact of NTS on the CPR quality percentage index, time of defibrillation, and time of airway clearance—total.

		CPR change	1st defibrillation change	Clearance change
NTS change	*rho*	0.703^∗∗^	−0.107	0.376
	*p*	0.002	0.684	0.137

*Note: rho*—Spearman’s correlation coefficient; *p*—significance.

^∗^ *p* < 0.05.

^∗∗^ *p* < 0.01.

^∗∗∗^ *p* < 0.001.

**FIGURE 2:**
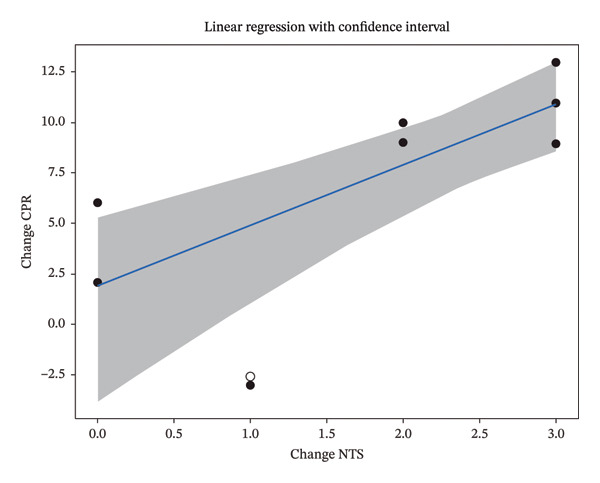
Correlation between change in NTS score and change in CPR score—study group.

In the intervention group (Group 1), a statistically significant correlation (*p* < 0.05) was noted between the NTS score and the change in the CPR result. An increase in NTS points was accompanied by a higher CPR score. However, no statistically significant correlation (*p* < 0.05) was noted between the NTS score and the 1st defibrillation time or airway clearance time (Table 4). To illustrate the change in CPR score, the correlation is also presented in Figure 2.

**TABLE 4:** Impact of NTS on the CPR quality percentage index, time of defibrillation, and time of airway clearance—study group.

		CPR change	1st defibrillation change	Clearance change
NTS change	*rho*	0.800^∗^	−0.004	0.287
	*p*	0.010	0.991	0.454

*Note: rho*—Spearman’s correlation coefficient; *p*—significance.

^∗^ *p* < 0.05.

^∗∗^ *p* < 0.01.

^∗∗∗^ *p* < 0.001.

In the control group (Group 2), no statistically significant correlation (*p* < 0.05) was noted between NTS score and the change in the CPR result or the 1st defibrillation time and airway clearance time. The hypothesis was rejected (Table 5).

**TABLE 5:** Impact of NTS on the CPR quality percentage index, time of defibrillation, and time of airway clearance—totals for the control group.

		CPR change	1st defibrillation change	Clearance change
NTS change	*r*	—	0.145	0.361
	*p*	—	0.733	0.380
	*rho*	0.645	—	—
	*p*	0.084	—	—

*Note: r*—Pearson’s correlation coefficient; *rho—*Spearman’s correlation coefficient*; p—*significance*.*

^∗^ *p* < 0.05.

^∗∗^ *p* < 0.01.

^∗∗∗^ *p* < 0.001.

## 4. Discussion

This research adopts a dual‐perspective approach: first, by analyzing the clinical impact of NTS on resuscitation quality, and second, by evaluating pedagogical methodologies for NTS training among paramedics. Evidence suggests that NTS serve as a critical framework for optimizing technical performance, including high‐quality chest compressions, rapid defibrillation, and advanced airway management. Collectively, these factors are essential for enhancing CPR efficacy and, ultimately, improving survival outcomes in SCA scenarios [14, 15]. A pivotal element of this research concerns the pedagogical framework and cognitive demands placed upon paramedics, who frequently serve as the primary providers of ALS in prehospital settings. While existing literature extensively documents NTS assessment among physicians—who typically lead hospital‐based resuscitation teams—there remains a critical knowledge gap regarding these competencies in paramedic‐led interventions. Consequently, our study offers a novel perspective by evaluating the impact of NTS within the specific context of prehospital Emergency Medical Services (EMS) [16]. In a seminal study of anesthesiologists, Flin and Patey [17] demonstrated that targeted NTS training for team leaders significantly mitigates procedural errors during life‐support interventions. In contrast, the present research design expands this scope by implementing NTS training for intact resuscitation teams—comprising three paramedics—thereby examining the collective impact of these competencies on team‐based performance. Although Graham et al. [18] suggest that brief NTS interventions may be insufficient to produce measurable improvements in clinical care, our findings indicate a different trend within the context of high‐fidelity simulation. Despite the inherent differences in study populations and instructional design, our data demonstrate that an intensive, four‐hour targeted training session is associated with a significant and observable enhancement in NTS proficiency among paramedics during simulated resuscitation. An additional critical factor in this study is the instructional methodology and the expertise of the facilitators. Training was delivered by certified ERC instructors with extensive experience in the pedagogical implementation of NTS within resuscitation teams. While the existing literature extensively documents the general impact of NTS on CPR quality, our research focuses on the efficacy of a standardized, expert‐led intervention specifically tailored for paramedic teams [19–22]. Notably, the majority of existing NTS research focuses on in‐hospital clinical teams, whose operational environment differs significantly from that of prehospital emergency crews. Resuscitation in the prehospital setting is characterized by a higher prevalence of unpredictable stressors, including limited personnel, uncontrolled environmental variables, and the presence of distressed family members at the scene—factors that necessitate a distinct approach to NTS application compared to hospital‐based interventions. This issue was previously explored by Krage et al. [23], who demonstrated that the introduction of exogenous stressors significantly impairs the quality of ALS. In contrast, our methodology deliberately omitted additional stress factors to isolate the baseline impact of NTS proficiency. Notably, even in the absence of induced stress, teams lacking formal NTS training exhibited substantial difficulties in executing core technical procedures, including high‐quality chest compressions, early defibrillation, and effective airway management. In a study by Peltonen et al. [24] investigating the impact of NTS on resuscitation quality, a positive correlation was observed between NTS scores and the proficiency of emergency medical procedures. Specifically, their analysis focused on the quality of chest compressions, ventilation, defibrillation, and rhythm recognition. However, the operational definition of “quality” in their assessment remains underspecified, making direct comparisons difficult [25]. Consequently, the present study prioritized objective, quantifiable metrics, specifically focusing on procedural duration and the chest compression expressed as a percentage. Our findings demonstrate that the disparities in chest compression quality between the NTS‐trained group and the control group were statistically significant. Furthermore, these performance gains positively correlated with the participants′ NTS assessment scores. While the significant improvement in chest compression quality is a promising finding, these results must be interpreted within the context of potential learning bias (the practice effect). Because all resuscitation teams were exposed to an identical SCA scenario during their second attempt, some of the observed improvements in technical performance could be partially attributable to increased familiarity with the simulation environment, the mannequin, and the specific clinical algorithm, rather than the NTS training alone. However, the inclusion of a control group in our design mitigates this confounding factor, allowing us to isolate the specific impact of the educational intervention. Furthermore, regarding methodological robustness, while the assessments were conducted by highly qualified GIC instructors using established evaluation matrices to maximize inter‐rater consistency, formal test–retest reliability coefficients were not independently calculated for this specific cohort across the repeated measures. To further validate these findings, future research should utilize alternative, clinically equivalent scenarios for postintervention assessments and incorporate rigorous psychometric evaluations of the measurement tools. Another methodological consideration is the use of a modified version of the Cooper et al. NTS assessment matrix. While the decision to exclude the “team morale” item was based on the practical challenges of reliably assessing this construct during a brief, high‐intensity 15‐min simulation, this modification presents certain limitations. Specifically, the omission of this parameter may have narrowed the content validity of the tool, potentially overlooking the emotional and social dynamics that influence team performance. Furthermore, utilizing a nonstandardized 10‐item version limits the direct comparability of our findings with other studies that employ the original 11‐item instrument. It should also be noted that the internal consistency and structural integrity of this modified scale were not independently validated for this specific study population. Future research should prioritize the use of fully validated, intact instruments or conduct rigorous psychometric evaluations when adaptations are necessary to ensure the continued reliability and sensitivity of the assessment. Interestingly, NTS proficiency did not demonstrate a statistically significant impact on defibrillation latency or airway management duration. This lack of correlation may be related to the inherent technical complexity and equipment‐specific requirements of these procedures. In the Polish EMS system, where this study was conducted, paramedics are highly trained professionals legally authorized to independently perform ALS interventions, including endotracheal intubation and defibrillation. Defibrillation necessitates meticulous team coordination and time‐intensive preparation. Similarly, advanced airway management involves a sequence of manual tasks—including device preparation, lubrication, and cuff inflation—all of which are time‐dependent variables that may remain independent of nontechnical performance [26]. Despite the inherent limitations of a controlled, simulation‐based environment, the findings of this study hold substantial translational relevance for real‐world prehospital EMS. Unlike structured in‐hospital settings, prehospital resuscitation frequently occurs in chaotic, unpredictable, and resource‐limited environments characterized by high cognitive overload and environmental stressors. In such scenarios, maintaining high‐quality chest compressions is a critical determinant of patient survival. Our observation that NTS proficiency positively correlates with chest compression quality suggests that structured training in communication, leadership, and task allocation directly supports the physical execution of core life‐saving interventions. While a high‐fidelity mannequin cannot fully replicate the complex psychological and environmental dynamics of an actual out‐of‐hospital cardiac arrest (OHCA), establishing a robust baseline of NTS in a simulated setting equips paramedics with the essential cognitive frameworks necessary to mitigate errors, streamline teamwork, and optimize clinical performance when transitioning to real‐world patient care.

## 5. Limitations

• The research was conducted in simulated laboratory conditions, which is the result of the impossibility of conducting an assessment in clinical conditions. The applied simulators fulfill the criteria for use in scientific research and provide the possibility for detailed monitoring and recording of the conducted procedures; however, they do not reflect the reality of clinical conditions.
• Modification of the original Cooper et al. NTS matrix by removing one item, which may affect the direct comparability of the obtained results with studies using the unmodified version of the tool.
• The study measures immediate educational outcomes; therefore, the long‐term retention of NTS and their impact on clinical practice over time were not assessed.
• Potential influence of the test effect, as the use of an identical simulation scenario in both the preintervention and postintervention assessments may have familiarized participants with the scenario and influenced the final outcome of the study.
• Lack of feedback after conducting the first attempt, although in this research, this was deliberate so as not to suggest any changes to research participants before the subsequent scenario. The only expected change was the use of the knowledge regarding NTS acquired during training.
• A second attempt was made in teams comprising the same people. To a certain degree, this attempt could have been based on self‐reflection and on the experiences from the first attempt—this was planned so as not to introduce additional distractors, while the teams were conducting resuscitation.
• Participants had limited time to become familiar with the defibrillator used during the research—most of the study participants declared that they were familiar with the equipment; however, it was impossible for the researchers to adapt the laboratory equipment on every occasion to the clinical conditions in which the study participants worked.
• Additionally, the analytical approach involved multiple statistical comparisons across different outcome variables (e.g., chest compression quality, defibrillation latency, and airway management duration). This inherently increases the risk of Type I errors (false positives). While traditional alpha‐level adjustments, such as the Bonferroni correction, can mitigate this risk, they were not applied in the present study. Given the exploratory nature of the research and the relatively small sample size, applying highly conservative corrections would overly inflate the risk of Type II errors (false negatives), potentially masking clinically relevant signals. Consequently, the unadjusted *p*‐values are reported. The statistically significant findings—particularly the correlation between NTS proficiency and chest compression quality—should, therefore, be interpreted as preliminary and hypothesis‐generating, emphasizing the need for future confirmatory trials.
• Another limitation of this study is the relatively small sample size (*n* = 51, comprising 17 resuscitation teams), which inherently restricts the statistical power of the analysis. While the sample was sufficient for an exploratory study to identify preliminary trends and statistically significant improvements in chest compression quality, the limited power increases the risk of Type II errors. Consequently, smaller but potentially clinically relevant differences in other metrics—such as defibrillation latency or airway management duration—might not have been detected. Therefore, these findings should be interpreted with caution, and future large‐scale, multicenter trials are warranted to confirm these results and enhance their generalizability.
• Similar research should be considered on a larger number of teams.

## 6. Conclusion

In conclusion, this exploratory study demonstrates that targeted NTS training is positively associated with enhanced nontechnical proficiency among paramedic resuscitation teams. Furthermore, higher NTS scores were observed alongside improved quality of chest compressions. While these findings suggest that integrating NTS training may play a valuable, supportive role in prehospital ALS, the observational nature of these correlations precludes definitive causal claims. Future large‐scale, multicenter trials are required to establish robust causal relationships and fully understand the long‐term clinical impact of NTS on specific procedural times.

## Author Contributions

Tomasz Ilczak: conceptualization, data curation, formal analysis, investigation, methodology, project administration, resources, software, supervision, validation, writing–original draft, and writing–review and editing. Michał Ćwiertnia: conceptualization, data curation, methodology, and supervision. Kacper Sumera: conceptualization, and methodology. Piotr Babik: conceptualization and methodology. Piotr Białoń: investigation. Mieczysław Dutka: formal analysis and investigation. Iwona Malinowska‐Lipień: validation and writing–review and editing. Magdalena Augustyn: data curation, validation, and methodology. Mateusz Majewski, Anna Lis, and Piotr Abramczyk: investigation. Piotr Leszczyński and Arkadiusz Stasicki: validation. Paweł Kukla: investigation and supervision. Wioletta Pollok‐Waksmańska: supervision. Joanna Trojak‐Piętka: investigation and software. Marek Kawecki: resources and supervision.

## Funding

This research received no funding.

## Disclosure

All authors edited and approved the final version of the manuscript. All authors have read and agreed to the published version of the manuscript.

## Ethics Statement

The research was conducted between April and May 2023 in the emergency medical laboratories of the University of Bielsko‐Biała in accordance with the Declaration of Helsinki. The research was approved by the Ethics Committee of UNIVERSITY OF BIELSKO‐BIALA (no. 2021/12/8E/9, approval date: 2021‐07‐12).

## Consent

This publication contains no individual data.

## Conflicts of Interest

The authors declare no conflicts of interest.

## Data Availability

The data that support the findings of this study are available from the corresponding author upon reasonable request.

## References

[1] Gjeraa K. , Jepsen R. M. , Rewers M. , Østergaard D. , and Dieckmann P. , Exploring the Relationship Between Anaesthesiologists Non-Technical and Technical Skills, Acta Anaesthesiologica Scandinavica. (2016) 60, no. 1, 36–47, 10.1111/aas.12598. 26272742

[2] Evans J. C. , Evans M. B. , Slack M. , Peddle M. , and Lingard L. , Examining Non-Technical Skills for Ad Hoc Resuscitation Teams: A Scoping Review and Taxonomy of Team-Related Concepts, Scandinavian Journal of Trauma, Resuscitation and Emergency Medicine. (2021) 29, no. 1, 10.1186/s13049-021-00980-5. PMC8642998 34863278

[3] Milatino Sgambati M. A. , d′Ercole A. , Cascio M. et al., Assessment of Nontechnical Skills During Resuscitation: Validation in the Italian Version of the TEAM, Simulation in Healthcare. (2024) 16, 10.1097/SIH.0000000000000807. 39007692

[4] Lloyd A. , Clegg G. , and Crouch R. , Dynamic Nurse Leadership in High-Pressure Situations, Emergency Nurse. (2015) 23, no. 3, 24–25, 10.7748/en.23.3.24.e1409. 26050780

[5] Peran D. , Sykora R. , Vidunova J. et al., Non-Technical Skills in Pre-Hospital Care in the Czech Republic: A Prospective Multicentric Observational Study (NTS Study), BMC Emergency Medicine. (2022) 22, no. 1, 10.1186/s12873-022-00642-4. PMC9107236 35562664

[6] Perkins G. D. and Nolan J. P. , Advanced Life Support Update, Critical Care. (2022) 26, no. 1, 10.1186/s13054-022-03912-6. PMC8957190 35337353

[7] Greif R. , Lockey A. , Breckwoldt J. et al., European Resuscitation Council Guidelines 2021: Education for Resuscitation, Resuscitation. (2021) 161, 388–407, Epub 2021 Mar 24. PMID: 33773831 10.1016/j.resuscitation.2021.02.016. 33773831

[8] Gaska K. , Pavlinec C. , Cebula G. , Pisarska-Adamczyk M. , and Szopa M. , Non-Technical Skills in Cardiopulmonary Resuscitation: Improvement and Evaluation of a New Course Introduced to the Curriculum at a Medical School in Poland in 2018 to 2019, Sage Open. (2023) 13, no. 4, 10.1177/21582440231205693.

[9] Siu J. , Maran N. , and Paterson-Brown S. , Observation of Behavioural Markers of Non-Technical Skills in the Operating Room and Their Relationship to Intra-Operative Incidents, The Surgeon. (2016) 14, no. 3, 119–128, 10.1016/j.surge.2014.06.005. 25022767

[10] Zausig Y. A. , Grube C. , Boeker-Blum T. et al., Inefficacy of Simulator-Based Training on Anaesthesiologists’ Non-Technical Skills, Acta Anaesthesiologica Scandinavica. (2009) 53, no. 5, 611–619, 10.1111/j.1399-6576.2009.01946.x. 19419355

[11] LeBlanc V. R. , Tabak D. , Kneebone R. , Nestel D. , MacRae H. , and Moulton C. A. , Psychometric Properties of an Integrated Assessment of Technical and Communication Skills, The American Journal of Surgery. (2009) 197, no. 1, 96–101, 10.1016/j.amjsurg.2008.08.011. 19101250

[12] Cortegiani A. , Russotto V. , Montalto F. et al., Use of a Real-Time Training Software (Laerdal QCPR®) Compared to Instructor-Based Feedback for High-Quality Chest Compressions Acquisition in Secondary School Students: A Randomized Trial, PLoS One. (2017) 12, no. 1, PMID: 28056076; PMCID: PMC5215847 10.1371/journal.pone.0169591. PMC5215847 28056076

[13] Cooper S. , Cant R. , Porter J. et al., Rating Medical Emergency Teamwork Performance: Development of the Team Emergency Assessment Measure (TEAM), Resuscitation. (2010) 81, no. 4, 446–452, Epub 2010 Feb 1. PMID: 20117874 10.1016/j.resuscitation.2009.11.027. 20117874

[14] Soar J. , Böttiger B. W. , Carli P. et al., European Resuscitation Council Guidelines 2021: Adult Advanced Life Support, Resuscitation. (2021) 161, 115–151, Epub 2021 Mar 24. Erratum in: Resuscitation. 2021 Oct;167:105-106. PMID: 33773825 10.1016/j.resuscitation.2021.02.010. 33773825

[15] Bucher J. , Feldman D. , and Joseph J. , Mistriaged Advanced Life Support Patients in a Two-Tiered, Suburban Emergency Medical Services System, Western Journal of Emergency Medicine. (2020) 21, no. 2, 449–454, PMID: 32191203; PMCID: PMC7081855 10.5811/westjem.2019.10.43885. 32191203 PMC7081855

[16] Farquharson B. , Cortegiani A. , Lauridsen K. G. , Yeung J. , Greif R. , and Nabecker S. , Education Implementation Team Task Force of the International Liaison Committee on Resuscitation ILCOR. Teaching Team Competencies Within Resuscitation Training: A Systematic Review, Res Publica. (2024) 19, PMID: 39006135; PMCID: PMC11239706 10.1016/j.resplu.2024.100687. PMC11239706 39006135

[17] Flin R. and Patey R. , Non-Technical Skills for Anaesthetists: Developing and Applying ANTS, Best Practice & Research Clinical Anaesthesiology. (2011) 25, no. 2, 215–227, 10.1016/j.bpa.2011.02.005. 21550546

[18] Graham J. , Hocking G. , and Giles E. , Anaesthesia Non-Technical Skills: Can Anaesthetists Be Trained to Reliably Use This Behavioural Marker System in 1 Day?, British Journal of Anaesthesia. (2010) 104, no. 4, 440–445, ISSN 0007-0912 10.1093/bja/aeq032. 20190257

[19] Andersen P. O. , Jensen M. K. , Lippert A. , and Østergaard D. , Identifying Non-Technical Skills and Barriers for Improvement of Teamwork in Cardiac Arrest Teams, Resuscitation. (2010) 81, no. 6, 695–702, Epub 2010 Mar 20. PMID: 20304547 10.1016/j.resuscitation.2010.01.024. 20304547

[20] Higham H. , Greig P. , Crabtree N. , Hadjipavlou G. , Young D. , and Vincent C. , A Study of Validity and Usability Evidence for Non-Technical Skills Assessment Tools in Simulated Adult Resuscitation Scenarios, BMC Medical Education. (2023) 23, no. 1, 10.1186/s12909-023-04108-4. PMC10007667 36906567

[21] Sanguanwit P. , Kulrotwichit T. , Tienpratarn W. , Athinartrattanapong N. , Trainarongsakul T. , and Angkoontassaneeyarat C. , Effect of Mini-Course Training in Communication and Teamwork on Non-Technical Skills Score in Emergency Residents: A Prospective Experimental Study, BMC Medical Education. (2023) 23, no. 1, PMID: 37491254; PMCID: PMC10369795 10.1186/s12909-023-04507-7. PMC10369795 37491254

[22] Hinski S. , Cooke N. J. , McNeese N. , Sen A. , and Patel B. , A Human Factors Approach to Building High-Performance Multi- Professional Cardiac Arrest Teams: Developing a Code Blue Team Performance Metric, Proceedings of the International Symposium on Human Factors and Ergonomics in Health Care. (2016) 5, no. 1, 68–71, 10.1177/2327857916051006.

[23] Krage R. , Zwaan L. , Tjon Soei Len L. et al., Relationship Between Non-Technical Skills and Technical Performance During Cardiopulmonary Resuscitation: Does Stress Have an Influence?, Emergency Medicine Journal. (2017) 34, no. 11, 728–733, Epub 2017 Aug 26. PMID: 28844039; PMCID: PMC5750366 10.1136/emermed-2016-205754. 28844039 PMC5750366

[24] Peltonen V. , Peltonen L. M. , Salanterä S. et al., An Observational Study of Technical and Non-Technical Skills in Advanced Life Support in the Clinical Setting, Resuscitation. (2020) 153, 162–168, ISSN 0300-9572 10.1016/j.resuscitation.2020.06.010. 32561474

[25] Sumera K. , Ilczak T. , Bakkerud M. et al., CPR Quality Officer Role to Improve CPR Quality: A Multi-Centred International Simulation Randomised Control Trial, Res Publica. (2024) 17, PMID: 38261942; PMCID: PMC10796959 10.1016/j.resplu.2023.100537. PMC10796959 38261942

[26] Ilczak T. , Ćwiertnia M. , Białoń P. et al., Endotracheal Tube Cuff Pressure- Comparison of the Two Filling Methods- Simulated Test, Prehospital and Disaster Medicine. (2021) 36, no. 4, 421–425, 10.1017/S1049023X21000406. 33928886

